# Index to the Transactions of the Epidemiological Society of London

**Published:** 1856-04

**Authors:** 


### INDEX.

Babington, Dr. B. G., report on cholera in the Black Sea and Baltic fleets in 1854, 89

Camps, Dr. W., fever at Cowbridge in 1853, 83

Cholera, prophylaxis of by mineral and vegetable acids, 10; in the Black Sea and Baltic fleets in the autumn of 1854, 89

Epidemiological Society, report of Council, 73; quarterly reports of, 24, 72, 87, 116

Fever at Cowbridge in 1853, 83

Geographical distribution of health and disease, 25

Johnston, Mr. A. K., geographical distribution of health and disease, 25

Kesteven, Mr. W. B., concurrent scarlet fever and measles, 80

Nurses, proposition to supply the labouring classes with, 1

Scarlet fever and measles, concurrent, 80

Sieveking, E. H., M.D., proposition to supply the labouring classes with nurses, 1

Tucker, Mr. J. H., prophylaxis of cholera by some of the mineral and vegetable acids, 10

